# Antiepileptic Effects of Hua‐Feng‐Dan Against Pentylenetetrazol‐Induced Seizures in Mice

**DOI:** 10.1155/bmri/5336509

**Published:** 2026-03-28

**Authors:** Wen-Ke Li, Bo Liu, Shang-Fu Xu, Jing-Shan Shi, Jin Li, Jie Liu

**Affiliations:** ^1^ Key Laboratory for Basic Pharmacology of Ministry of Education and Joint International Research Laboratory of Ethnomedicine, Zunyi Medical University, Zunyi, China, zmc.edu.cn; ^2^ Key Laboratory of Infectious Disease and Biosafety, Guizhou Provincial Department of Education, Zunyi Medical University, Zunyi, China, zmc.edu.cn

**Keywords:** brain RNA-seq, gut microbiota, Hua-Feng-Dan, pentylenetetrazol (PTZ), seizure score

## Abstract

Hua‐Feng‐Dan is a Chinese medicine in the treatment of epilepsy and neurodegeneration. The prior studies demonstrated its protection against neuroinflammation, dopaminergic neuron loss, and in the treatment of ischemic stroke, but its anticonvulsive efficacy against epilepsy remains elusive. The aim of this study was to examine antiepileptic effects of Hua‐Feng‐Dan recipes and the mechanism of protection. Pentylenetetrazol (PTZ)‐induced kindling mouse model (35 mg/kg, ip on alternate days × 10) was used to evaluate the antiepileptic effects of Hua‐Feng‐Dan recipes. Mice were given original, reduced, and nonfermented Hua‐Feng‐Dan via feed at clinical dose (0.5 g/kg) and diazepam was used as a positive control. The seizure scores were recorded after each PTZ injection. At the end of the experiments, the brain and colon content were collected for RNA‐Seq and 16S rRNA‐Seq, respectively, followed by bioinformatics and qPCR verification. Hua‐Feng‐Dan recipes were effective against PTZ‐induced seizures, with original the best and half of reduced (0.25 g/kg) ineffective. PTZ‐induced aberrant gene expressions in the brain were ameliorated by Hua‐Feng‐Dan recipes to various extents. Ingenuity Pathway Analysis of differentially expressed genes revealed that PTZ‐altered canonical pathways and upstream regulators were attenuated by Hua‐Feng‐Dan treatments. qPCR verified the expression of early immediate genes, proinflammatory mediators, transporters and apoptosis genes. PTZ‐disrupted gut microbiome was also ameliorated by Hua‐Feng‐Dan recipes, and the modulation of *Lachnospiraceae* and ASV230 was consistent with the literature. Hua‐Feng‐Dan recipes were effective against PTZ‐induced kindling mice. The mechanisms appeared to be related to the modulation of brain gene expression and gut microbiota.

• Hua‐Feng‐Dan (HFD) recipes were effective against PTZ‐induced seizures in mice.
• HFD ameliorated abnormal brain gene expressions in PTZ kindling mice via RNA sequencing (RNA‐seq).
• IPA of DEGs revealed improved canonical pathways and upstream regulators.
• 16S rRNA‐seq showed microbiome modulation by HFD in PTZ kindling mice.
• qPCR verified selected brain genes and bacterial ASVs.

## 1. Introduction

HFD was created in 1644, and reputed for its therapeutic effects in the treatment of various brain diseases, including stroke recovery, epilepsy, and facial nerve paralysis, due to its “calm the wind” and anticonvulsion properties [1]. HFD has been patented as a traditional medicine for brain diseases in 2004 by China Intellectual Property Administration, with Chinese FDA approval (GYZZ Z2002646).

Original HFD recipe contains herbs, minerals, and others (*Gastrodia elata* Bl.; *Nepeta tenuifolia*; *Croton tiglium* L.; *Atractylodes lancea* (Thumb.) DC.; *Typhonium giganteum* Engl.; *Blumea balsamifera* (L.) DC.; *Acorus tetarinowii* Schott; *Perilla frutescens* (L.) Britt; *Moschus berezovskii* Flerov; *Buthus martensii* Karsch; *Bombyx mori* Linnaeus, realgar, cinnabar, Borax) [1, 2]. The plant names have been checked with http://www.worldfloraonline.org (access data 9‐15‐2025). For safety consideration, cinnabar and realgar are reduced from 10% to 3% and termed “Reduced Hua‐Feng‐Dan” [3, 4]. Cinnabar (*α*‐HgS) has been used in traditional medicines for 2000+ years, and is still included in 56 formulae of pharmacopeia of China, including the famous *Angong Niuhuang* Wan that also contains 10% cinnabar and 10% realgar [5]. Cinnabar is a classical sedative‐hypnotic drug included in many anticonvulsant formulae to play critical roles [6]. However, for safety concerns, both cinnabar and realgar are reduced from 10% to 3% and termed “Reduced” HFD.

The phytochemical analysis using GC–MS, 44 volatile and 50 liposoluble components in HFD was profiled [7]. Recently, UPLC‐Q‐TOF‐MS/MS detected 51 different components between fermented and nonfermented HFD [8]. HFD contains 18% fermented “*Yaomu*” that plays an important role in the pharmacological effects of HFD [7–9] “*Yaomu*” was added in raw without fermentation termed “Nonfermented” HFD. The full botanical plant names, batch number, and the ratio of the ingredients can be found in a recent publication [8].

The prior studies have demonstrated that HFD protects against Parkinson′s disease models in mice [10] and in rats [3]. There is a relationship between movement disorders and antiseizure drugs such as ethosuximide and valproate [11], and the antiepileptic effect of HFD is one of its major clinical applications. Recently, it was reported in a clinical study that HFD in combination with sodium valproate is effective in the treatment of epilepsy patients [12]. There is a gap between the clinical application of HFD and its experimental basis, and the current study aimed to fill the gap.

Among experimental epilepsy animal models, pentylenetetrazol (PTZ)‐induced kindling of epilepsy is the most widely used model to understand the pathophysiology of epilepsy, which is defined as a chronic disease involving repetitive seizures [13]. In comparison to acute seizure induced by a single high dose of PTZ, chronic seizures induced by subconvulsive stimuli with repetitively and intermittently injection (usually 10 times) will lead to the generation of tonic–clonic seizure more relevant to clinical manifestations [14]. The PTZ‐kindling model resembles many clinical features of epilepsy including chronic repetitive seizures and neuronal damage [15], but also produces abnormal expression of immediate early genes in the brain [16] and gut dysbiosis [17], and is frequently used to evaluate novel antiepileptic medicines [17–20].

A recent study also demonstrated that HFD is effective against cerebral ischemia stroke in rats [2] in addition to HFD effects against Parkinson′s disease [3, 10]. These studies point towards the modulation of gut microbiota as one of the protective mechanisms [2, 3, 10]. The brain–gut‐microbiota axis could play a role in the neuroprotective effects of HFD. Our prior work using RNA‐Seq revealed the beneficial effects of HFD and “fermented Yaomu” in the liver [7], and RNA‐Seq analysis of the brain could be useful in elucidating the molecular mechanisms.

In the present study, the PTZ kindling mouse model was used to examine antiepileptic effects of HFD recipes, and to test the hypothesis that the beneficial effects of HFD are mediated through the modulation of aberrant brain gene expression and gut dysbiosis, using diazepam as the positive control. Since diazepam is a well‐known anxiolytic drug, the anxiolytic effects of HFD recipes in normal mice without PTZ challenge have also been examined as one of the anticonvulsant mechanisms [21]. The results obtained demonstrated antiepileptic effects of HFD and the potential mechanisms.

## 2. Materials and methods

### 2.1. Materials

HFD original (containing 10% cinnabar and 10% realgar), reduced (containing 3% cinnabar and 3% realgar), and nonfermented (the Guide Drug “Yaomu” was not fermented for 3 months) were provided by HFD Pharmaceutical Co. (Zunyi, China). Diazepam is a medicine of the benzodiazepine family from Guiyang Pharmaceutical Co. (Guiyang, China). PTZ was from Sigma‐Aldrich (St. Louis, Missouri). All other chemicals were of reagent grade.

### 2.2. Animals and Drug Treatment

Adult male Kunming mice (20–22 g) were purchased from the Experimental Animal Center of Army Medical University (Chongqing, China) (Certificate No. SCXK, 2012‐0011). Animals were maintained in the SPF‐grade facilities of the Key Laboratory of Basic Pharmacology of Ministry of Education, Zunyi Medical University, at 20^°^C ± 2^°^C, 12 h dark: 12 h light, and had feed and drinking water ad libitum. The experimental protocol followed the Chinese Animal Use and Welfare Guidelines and was approved by the Institutional Animal Care and Use Committee of Zunyi Medical University (ZMU21‐2403‐054).

### 2.3. Experimental Design

After 1 week of acclimatization, mice were given various HFD recipes via diet based on clinical dosage as previously described [3, 10]. Based on feed consumption, approximate doses were 0.5 g/kg for HFD original, reduced, nonfermented, and PTZ (*n* = 10) in the 1st experiments. In the 2nd experiment (*n* = 8–9) with adding half of reduced (0.25 g/kg, *n* = 8) termed as “Reduced_Low” to assess dose‐dependent effects. HFD recipes were pretreated 3 days before 1st PTZ injection and lasted till the 10th PTZ injection (20 days), and mice were euthanized 30 min after the last PTZ injection. Controls were fed normal rodent chow. All diets were prepared by Jiangsu Xietong Biology (Nanjing, China). PTZ was freshly prepared in saline and injected (35 mg/10 mL/kg, ip) on alternative days for a total 10 injections [14]. In contrast to acute seizure induced by a single high dose (80 mg/kg and above), the 10 injections of subconvulsive dose induce tonic–clonic seizures. Diazepam was dissolved in saline at the dose of 2 mg/kg, po. 30 min before PTZ injection. PTZ was injected at 9:00 am in the morning and behaviors were observed after injection for 30 min [13], The criteria for seizure scores were Grade 1, immobilization; Grade 2, partial myoclonus; Grade 3, whole‐body myoclonus with vertical tail; Grade 4, rearing, tonic seizure; and Grade 5, tonic–clonic seizures [14].

### 2.4. Brain RNA‐Seq

Transcriptome analysis of brain was conducted as previously described [22]. Briefly, total RNA was extracted using RNAiso Plus (TaKaRa Bio Inc., Dalian, China) following the instructions provided by the manufacturer. NanoDrop was used to ascertain RNA quality and quantity. Samples were sent to Shanghai Majorbio Bio‐pharm Biotechnology Co., Ltd. (Shanghai, China) to verify RNA quality and perform RNA purification, reverse transcription, library construction and sequencing using the Illumina NovaSeq 6000 platform.

### 2.5. Bioinformatic Analysis

All the RNA‐Seq reads (57,000) were imported into Partek Flow (Qiagen, Germantown, Maryland, United State) for analysis. Differentially expressed genes (DEGs) analysis (DEGs) was determined with the DESeq2 method under the criteria of *p* < 0.05.

DEGs were subjected to two‐dimensional, hierarchical complete linkages to generate Cdt files, followed by TreeView (Version 1.6) for visualization.

DEGs were also uploaded into Ingenuity Pathway Analysis (IPA) (Qiagen, Redwood City, California, United States) for core analysis and comparative analysis. Canonical pathways and upstream regulator analyses were performed. The *Z*‐scores were used to evaluate changes between PTZ_vs_Control and PZT + Treatment_vs Control groups.

### 2.6. Microbial 16S rRNA Sequencing

Fresh colon content was collected and stored at –80°C until analysis. Bacterial DNA was extracted by stool DNA kit (R1200; Solarbio, Beijing, China) according to the manufacturer′s instructions. DNA quality and concentration were measured using NanoDrop ND‐2000 (Thermo Fisher Scientific Inc., United States) to ascertain 260/280 > 1.8, concentration >20 ng/*μ*L. Samples were sent to Shanghai Majorbio Bio‐Pharm Biotechnology Co., Ltd. (Shanghai, China) for quality check and 16S rRNA sequencing.

The Majorbio Cloud provided a one‐stop, comprehensive bioinformatic platform for multiomics analyses [23]. The Illumina MiSeq fastq reads were imported into the QIIME2 (quantitative insights into microbial ecology) pipeline, and the optimized data were processed using the sequence denoising method (DADA2). The ASV (amplicon sequence variant) represents the sequence and abundance information and was used for analysis. Alpha diversity was used to evaluate the microbial community diversity, including Chao, Shannon, and Simpson indices. Beta diversity was used to visually evaluate the difference of bacterial communities between control and treatment groups by principal coordinate analysis (PcoA) based on operational taxonomic units (OUT) levels.

### 2.7. Real‐time qPCR

Total RNA was reverse transcribed using the PrimerScript RT kit (TaKaRa Biotechnology Co., Ltd., Dalian, China) to convert RNA to cDNA. Bacterial DNA was directly used for qPCR. The primers were designed by Primer3 (Version 4) and synthesized by Sangon Biotech (Shanghai, China) (Table S1). The PCR reaction was carried out as previously described [22] with a CFX 96 real‐time fluorescence quantitative PCR instrument (Bio‐Rad, Hercules, California, United States) using the iTaq Universal SYBR Green Supermix (Takara Bio, Japan). The relative gene expression levels were normalized with *Gapdh* of each sample and calculated using the 2^−△△Ct^ formula. Total bacteria (*Universal*) were used as internal control for bacterial ASV expression.

### 2.8. Statistics

The PTZ‐induced seizure scores were analyzed using one‐way repeated ANOVA and Shapira–Wilk normality test, followed by Bonferroni *t*‐test; DEGs and ASVs from RNA‐seq and 16S rRNA‐Seq were analyzed by DESeq2; qPCR data were expressed as mean ± SEM, and subjected to one‐way ANOVA, followed by Dunn′s multiple range tests. *p* < 0.05 was considered significant.

## 3. Results

### 3.1. HFD Alleviated PTZ‐Induced Kindling in Mice

**Figure 1:**
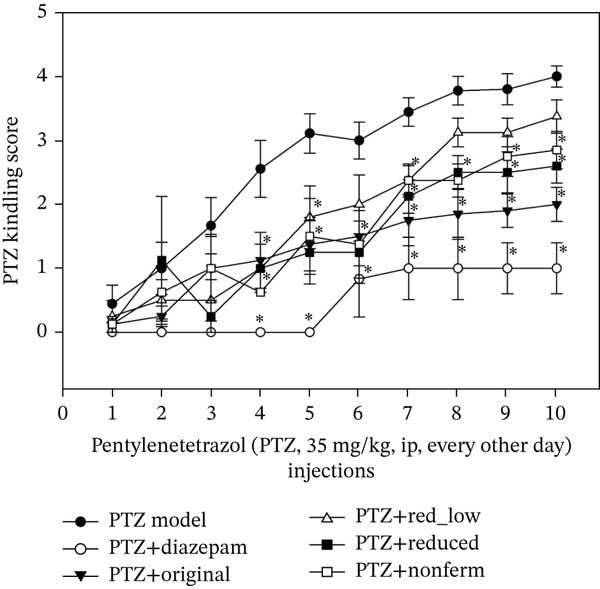
Effects of Hua‐Feng‐Dan on PTZ‐induced kindling in mice. Mice were pretreated with different Hua‐Feng‐Dan diets (0.5 and 0.25 g/kg for low) for 3 days and then received freshly prepared PTZ (35 mg/kg, ip) every other day for 10 injections, and mice continued on Hua‐Feng‐Dan diets till the end of experiments. Diazepam (2 mg/kg, po) was administered 30 min before each PTZ injection as positive control. Seizure scores were recorded after PTZ injection in the 2nd experiment [14]. Data are mean ± SEM (*n* = 8–9).  ^∗^Significantly different from PTZ mice *p* < 0.05.

PTZ is a gamma‐aminobutyric acid (GABA) Type A (GABA_A_) receptor antagonist widely used for producing a kindling model of epilepsy [13]. PTZ is administered chronically at a subconvulsive dose (35 mg/kg, ip) for 10 times at 48 h intervals to reach fully kindling. No mortality occurred in the experiments. Seizure scores were evaluated after each PTZ injection based on criteria described in detail in Methods [14]. The effects of HFD recipes (0.25–0.5 g/kg in feed) and the positive drug diazepam (2 mg/kg, ip, 30 min before PTZ injection) on PTZ seizure scores were recorded (Figure 1) during the 2nd experiment (Only the fully seizure scores after 10 PTZ injections were recorded during the 1st experiment).

After the PTZ model mice were fully kindled (after 10 injections), the seizure scores were calculated combining the 1st and 2nd experiments, and the results are shown in Table 1 using one‐way repeated ANOVA, followed by Shapiro–Wilk normality test and Bonferroni *t*‐test for multiple group comparisons. Compared with PTZ model, diazepam is very effective with *t*‐value of 13.382, followed by HFD original (*t*‐value 11.564), Reduced_H (0.5 g/kg, *t*‐value 7.214), nonfermented (*t*‐value 7.118), whereas Reduced_L (0.25 g/kg) was ineffective (*t*‐value 0.342, p 1.000). In addition, HFD original was better than Reduced_H (*t*‐value 4.029, *p* < 0.003) and nonfermented (*t*‐value 4.265, *p* < 0.001), despite all recipes being effective at the clinical dose (0.5 g/kg).

**Table 1:** Effects of Hua‐Fend‐Dan on seizure scores after 10 PTZ injections.

Group	*n*	Grade 0	Grade 1	Grade 2	Grade 3	Grade 4	Grade 5	Mean	t_vs_PTZ	*p*
Control	13	13	0	0	0	0	0	0.000	17.862	< 0.001
PTZ model	19	0	0	0	3	11	5	4.105		
PTZ + diazepam	16	2	9	2	3	0	0	1.375	13.382	< 0.001
PTZ + original	18	2	3	7	5	1	0	2.000	11.564	< 0.001
PTZ + reduced_L	8	0	0	1	4	3	0	3.250	0.342	1.000
*P* *T* *Z* + *r* *e* *d* *u* *c* *e* *d*_*H*	17	2	2	3	5	3	2	2.647	7.214	< 0.001
PTZ + nonferment	18	1	2	5	4	4	2	2.778	7.188	< 0.001

*Note:* Data are in combination of the 1st (2023) and 2nd (2024) experiments (*n* = 8–19). The criteria for seizure scores are detailed in Methods based on criteria in the literature [14].

### 3.2. RNA‐Seq Analysis of DEGs

To explore the mechanism of HFD mediated protection against PTZ kindling, brain RNA‐seq was conducted. RNA‐seq generated 57,180 reads, which were imported into Partek Flow (Qiagen, Germantown, Maryland) for analysis. Figure 2a shows DEGs by DESeq2 analysis. Under the criteria of *p* < 0.05, PTZ_model versus control had 251 genes up and 97 genes down; PTZ_Diazepam versus control had 101 up and 137 down genes; PTZ_Original versus control had 280 up and 91 down genes; PTZ_Reduced_L versus control had 75 up and 60 down genes, PTZ_Reduced_H versus control had 138 up and 55 down genes, and PTZ_Nonfermented versus control had 215 up and 57 down genes. Hierarchical clustering of DEGs is shown in Figure 2b. Please note that the clusters of PTZ decreased 42 genes (e.g., Gm29394, −6.9, Oxt, −4.2, Cdhr1, −3.3) were not seen in the treatment groups (the 1st arrow, line 220–262), and PTZ increased 75 genes (e.g., Il17rc, 4.2, Ptgs2, 3.8, Mrm1. 2.4) were normal in the treatment groups (the 2nd arrow, line 677–752). The 2‐D cluster genes were provided in Table S2.

Figure 2RNA‐seq analysis. Brains were collected at the end of experiments and total RNA was extracted for RNA‐seq analysis (*n* = 4, 2 from the 1st and 2 from the 2nd experiments). (a) Differentially expressed genes (DEGs) under *p* < 0.05; (b) clustering heat map of DEGs. Red indicates upregulation, whereas blue indicates downregulation.

**Figure:**
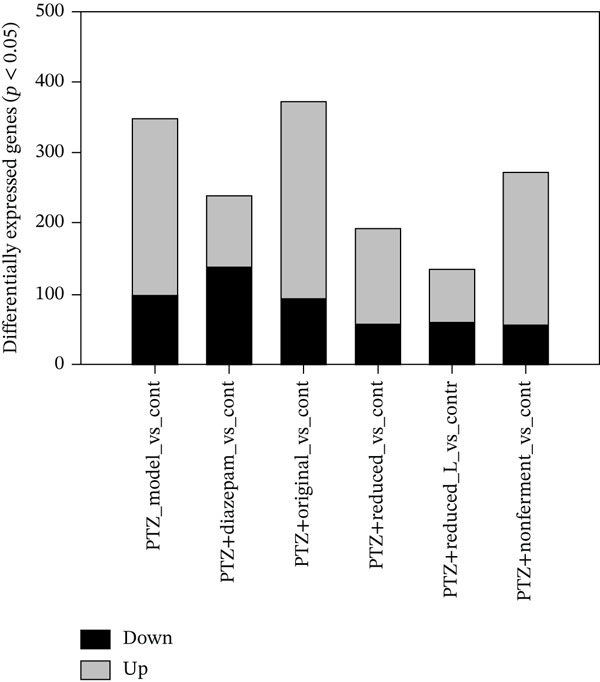
(a)

**Figure:**
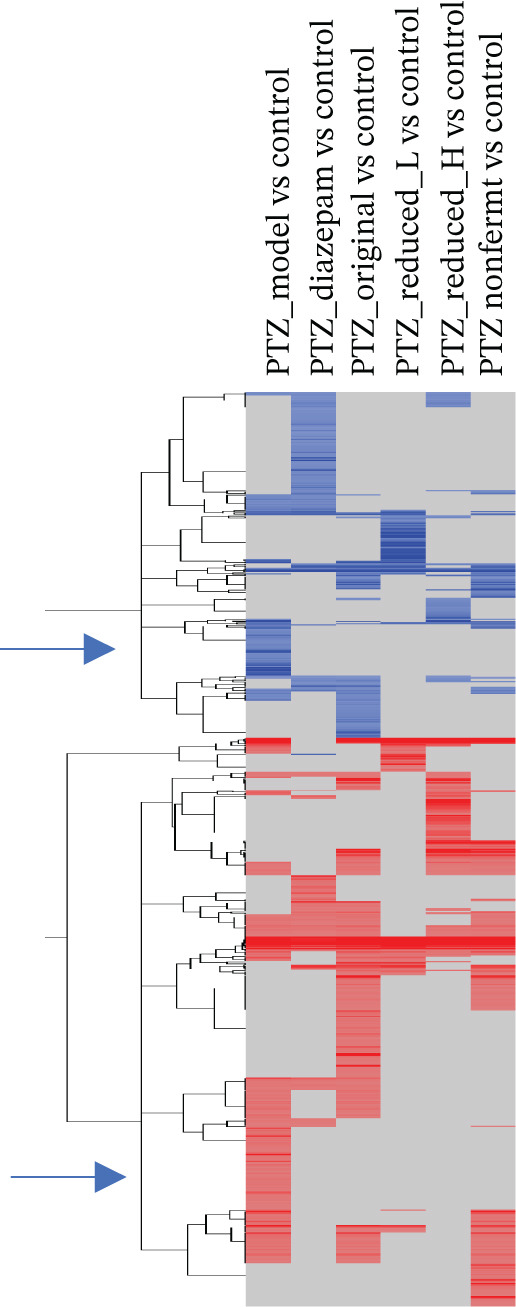
(b)

### 3.3. IPA of DEGs

The Top 15 canonical pathways of IPA (Figure 3a) revealed that PTZ mainly affected transcriptomes (eukaryotic translation initiation, ribosomal and rRNA processing, and protein ubiquitination), mitochondria (oxidative phosphorylation, electron transport, mitochondrial dysfunction, Complex I biogenesis), and cellular adaptive responses (nonsense‐mediated decay, neutrophil extracellular trap, granzyme A and EIF2 signaling, Parkinson′s signaling and hematoma resolution), with 12 upregulations and 3 downregulations. The color intensity of the PTZ model (1st column) was attenuated following diazepam and HFD treatments to various degrees. The Top 15 upstream regulators of IPA (Figure 3b) revealed that PTZ affected multiple signaling pathways (RICTOR/mTOR, sirolimus, CREB1, and LARP1), hormonal homeostasis (LH and NGF), immune function (BCR, CD40, and TCR), oncogene (MYC and MYCN) and cell metabolism and function (CPT1B, MLXIPL, TEAD1 and kainic acid), with 11 upregulations and 4 downregulations. Again, the color intensity of the PTZ model (first column) was attenuated to various extent by diazepam and HFD recipes.

Figure 3Ingenuity pathway analysis (IPA). (a) Top 15 canonical pathways based on *Z*‐scores. (b) Top 15 upstream regulators based on *Z*‐scores. Red indicates the upregulation and blue indicates the downregulation as compared with the normal control.

**Figure:**
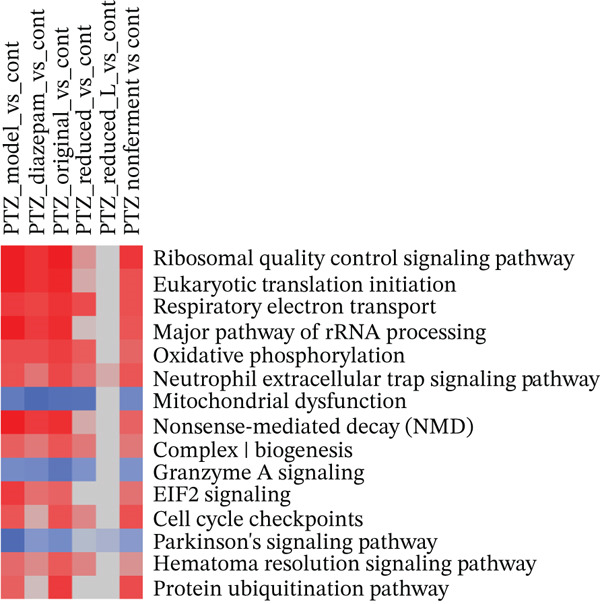
(a)

**Figure:**
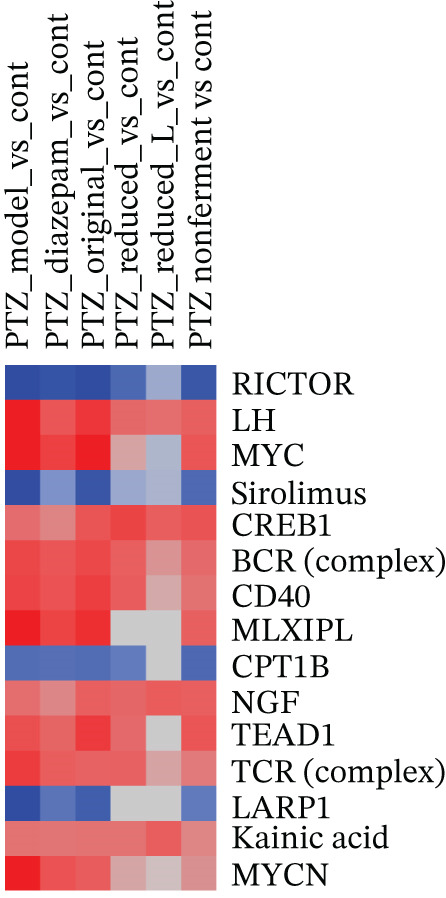
(b)

**Figure 4:**
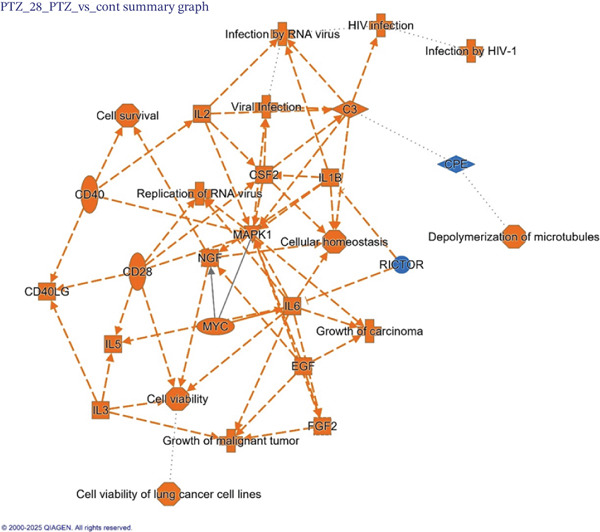
Ingenuity pathway analysis (IPA) of PTZ. Graphic summary of major molecules altered by PTZ versus normal controls. Color in orange indicates upregulation, whereas color in Blue indicates downregulation. The shapes of the nodes imply the major molecules or biological effects.

IPA of DEGs (*p* < 0.05) from PTZ model versus control generated a graphic summary (Figure 4). In this network connections, cellular homeostasis and MAPK1 signaling were activated by inflammatory mediators (IL‐6, IL‐1*β*), immune molecules (IL‐2, IL‐3, IL‐5, CD28, and CD40), growth factors (NGF, EGF, FGF2, and CSF2), and functional molecules (MYC and C3), whereas the RICTOR (member of mTORC2 complex) signaling pathway and the neuroprotective molecule CPE (carboxypeptidase E) [24] were decreased.

### 3.4. qRT‐PCR Analyses

**Figure 5:**
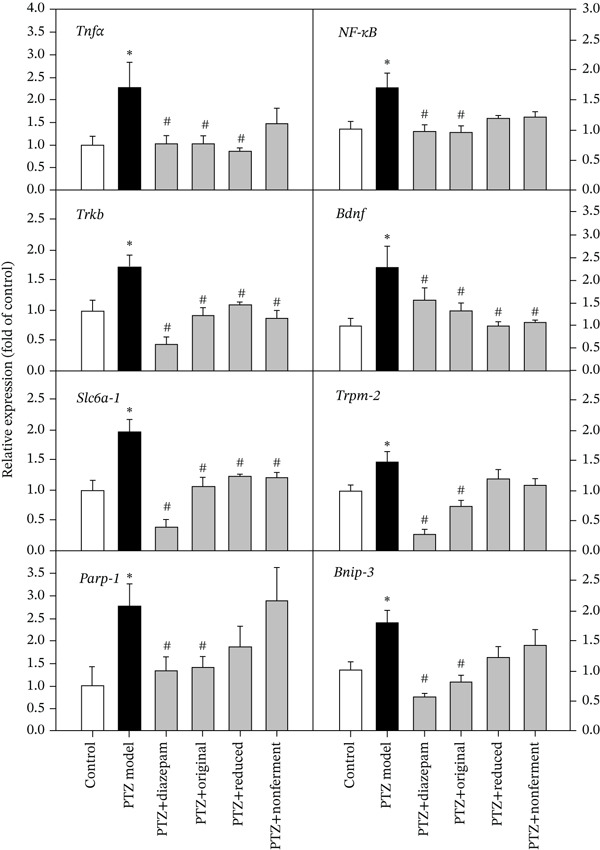
qPCR analysis of selected immediate early genes. Total RNA from the 1st experiments (*n* = 8) was subjected to qPCR verification. Data are mean ± SEM, ∗PTZ versus control, *p* < 0.05; #Treatment versus PTZ, *p* < 0.05.

According to the graphic summary of PTZ model and the literature on PTZ‐induced DEGs, the following category of genes was selected for qPCR analysis: (1) inflammation [25], (2) BDNF/TrkB pathway [26], (3) GABA transporter and Trpm2 ion channel [26, 27] and apoptosis‐related Parp1 and Bnip3 [28]. Figure 5 showed the upregulation of these genes in PTZ kindling mice, and all were attenuated by diazepam and original HFD. Reduced HFD was also effective for *Tnfα, TrkB, Bdnf*, and *Slc6a1*, whereas nonfermented HFD was effective for *TrkB, Bdnf*, and *Slc6a1*. Half dose of reduced HFD was also effective for *Tnfα, TrkB, Bdnf,* and *Slc6a1.*

**Figure 6:**
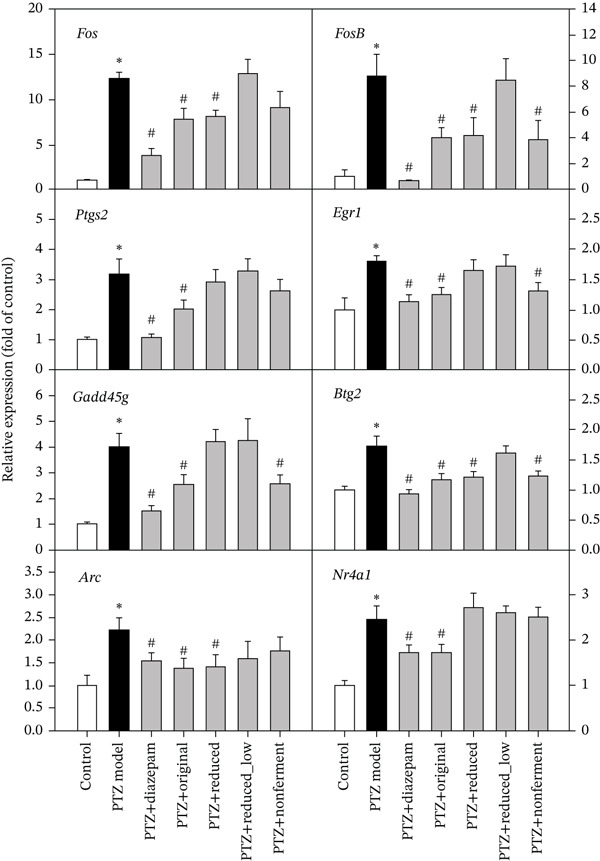
qPCR analysis of selected immediate early genes. Total RNA from the 1st and 2nd experiments (*n* = 10) was subjected to qPCR verification. Data are mean ± SEM, ∗PTZ versus control, *p* < 0.05; #Treatment versus PTZ, *p* < 0.05.

Based on the hierarchical clustering of DEGs shown in Figure 2, eight immediate early genes were selected [16] for qPCR verification (Figure 6). As expected, chronic PTZ‐induced kindling increased the expression of immediate early genes *Fos, FosB, Ptgs2, Egr1, Gadd45g, Btg2, Arc*, and *Nr4a1*. All of them were significantly attenuated by diazepam and original HFD, and some of them were also alleviated by reduced and nonfermented HFD (*Fos, FosB, Egr1, Gadd45g*, *Btg2,* and *Arc*), whereas the half dose of reduced HFD (0.25 g/kg) was less effective.

### 3.5. Effects of HFD on Gut Microbiome Composition in PTZ Kindling Mice

Emerging evidence indicates that the gut‐microbiota‐brain axis is closely related to brain disorders, including epilepsy [29]. Modulation of gut microbiota mediates the antiepileptic effects of drugs in the PTZ kindling model [19]. The composition of the gut microbiome, *α*‐diversity, and *β*‐diversity are important parameters to evaluate drug effects in the PTZ kindling model [18]. HFD has been shown to exert neuroprotective effects via modulating the gut microbiome in LPS plus MPTP model mice [10], in LPS plus rotenone‐treated rats [3], and in middle cerebral artery occlusion (MACO) rats [2], and whether the antiepileptic effects of HFD recipes are mediated through gut microbiota modulation was examined.

In the present study, the 15960 ASV reads belong to 15 phyla, and the Top 8 phyla were presented in Figure 7a, the composition percentage of Bacteroidota (B)/Firmicutes (F) ratio varied among groups (1.38, 1.29, 1.11, and 1.69 for control, PTZ, diazepam, and original, respectively), but they are not statistically significant. The Top 20 out of 32 families were selected to make the heat map (Figure 7b) comparisons. However, there were no significant alterations in *α*‐diversity and *β*‐diversity.

Figure 7Gut microbiota composition. Bacterial DNA was isolated from colon content and subjected to 16S rRNA‐seq (*n* = 4) from the 2nd experiment. (a) Microbiota composition at the major phylum level; (**b)** the composition at the family level, color intensity indicates the abundance.

**Figure:**
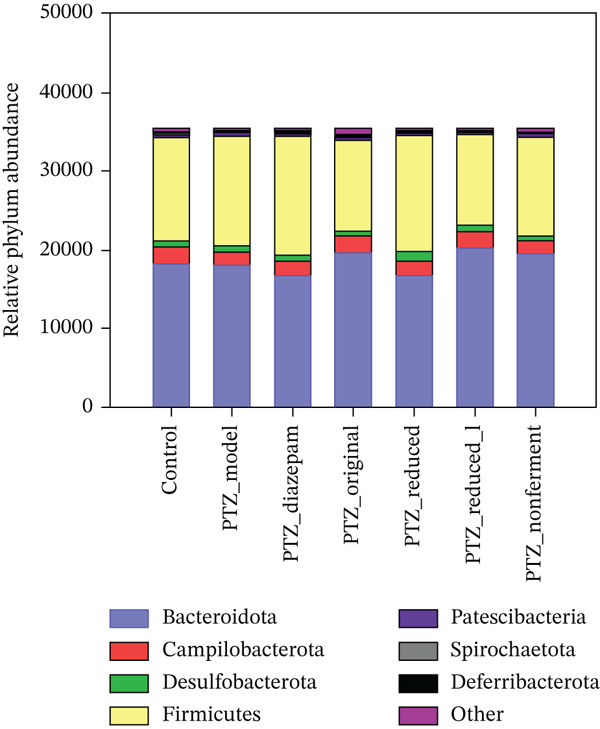
(a)

**Figure:**
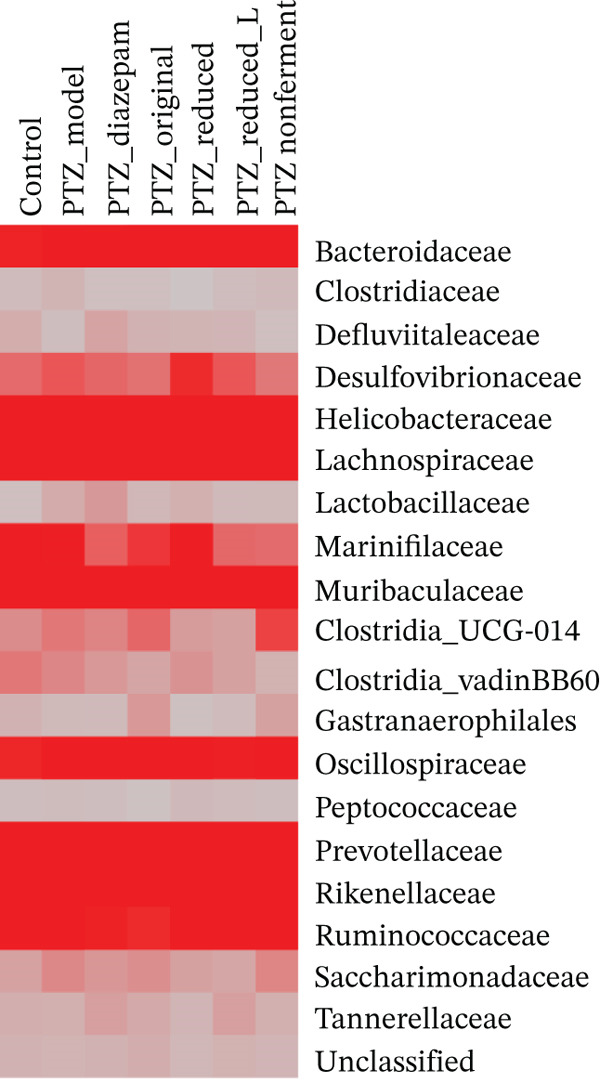
(b)

**Figure 8:**
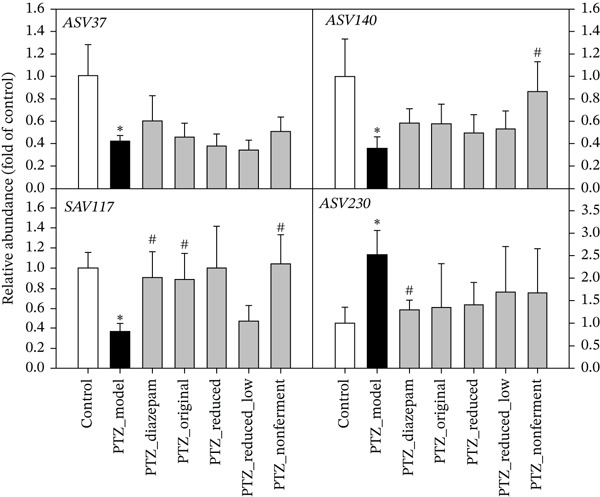
qPCR verification of selected ASVs. qPCR on selected ASVs with specific primers (Table S1). Data are mean ± SEM (*n* = 8) from the 2nd experiment. ∗PTZ versus control, *p* < 0.05; #Treatment versus PTZ, *p* < 0.05.

An ASV could detect bacterial DNA variation. qPCR was further used to examine selected 4 ASVs (Figure 8). PTZ increased ASV230 and decreased ASV37, ASV140, and ASV117. Diazepam and HFD could ameliorate such microbiome disruption to various extent. Even nonfermented HFD could reverse PTZ‐decreased ASV140 and ASV117, but the half of Reduced_Low was ineffective compared with PTZ_Model.

## 4. Discussion

The present study clearly demonstrated the antiepileptic effects of HFD in the PTZ kindling mouse model. Original, reduced, and nonfermented recipes were effective against seizures, with original better, whereas the half dose of reduced recipe was ineffective. Brain RNA‐seq revealed DEGs among groups, and IPA of DEGs revealed PTZ altered canonical pathways and upstream regulators, which were ameliorated by HFD treatments. qPCR verified RNA‐seq results, and PTZ‐increased the expression of immediate early genes, inflammation mediators, transporters, and apoptosis genes were attenuated by diazepam and HFD recipes to various extents; gut microbiome analysis via 16S rRNA‐seq showed that PTZ‐induced dysbiosis could be ameoliated by HFD treatments. This study filled the gap between clinical application of HFD against epilepsy and pharmacological basis.

PTZ is a GABA_A_ receptor antagonist, and the PTZ kindling mouse model is widely used to mimic chronic, repetitive seizures in epilepsy patients [13, 14]. PTZ kindling is ideal to evaluate the antiepileptic effects of traditional medicines [17, 19]. The 2nd experiment recorded the time‐course of PTZ‐induced seizures and combined with the 1st experiment to make the statistical analysis at the full‐kindled phase after 10 PTZ injections (Table 1). The Bonferroni *t*‐test values in comparison with the PTZ model were 13.382, 11.564, 7.214, and 7.118 for diazepam, original, reduced, and nonfermented HFD, respectively, indicating therapeutic efficacy in the HFD recipe in the treatment of epilepsy. However, original HFD was better than reduced and nonfermented, indicating the role of cinnabar and “*Yaomu*” fermentation against PTZ‐induced seizures.

Cinnabar is well‐known for its sedative and anticonvulsant effects [5, 6]. Recent studies found that cinnabar is essential in Angong Niuhuang Wan to assist therapeutic effects against ischemic shock [30]; similarly in *Shang-He-Jie-Gu* Tablet, cinnabar is necessary to reduce neurotoxicity of Strychni Semen [31]. Cinnabar is an important mineral drug not only for sedative and anticonvulsant effects, but also could act as nano‐delivery carries in multifunctional delivery systems [32]. Although both original and reduced HFD were effective against PTZ‐induced chronic seizures, original was better in the present study, consistent with the anxiolytic effects in normal mice. Indeed, reducing or removing cinnabar from HFD recipe compromised its antineuroinflammation effects [3, 4] and attenuated its modulating effects on gut microbiota [3]. The necessity of including 10% cinnabar in the Original HFD recipe still requires further clarification and investigation.

Brain RNA‐seq revealed PTZ‐induced DEGs, which were ameliorated following diazepam and HFD treatments as visualized in 2‐D hierarchical clustering of DEGs. The DEGs were further uploaded into IPA for bioinformatics analysis. IPA of canonical pathways (Figure 3a), upstream regulators (Figure 3b), and graphic summary of PTZ‐induced changes (Figure 4) revealed molecular events in PTZ‐induced kindling mouse brain and the therapeutic effects of HFD. It is worth mentioning that the PTZ‐induced alterations were attenuated by HFD in the same direction as diazepam, though to various extents. In canonical pathways, transcriptome regulations, mitochondria functions, inflammation signaling, adaptive responses, and repair mechanisms are predominated. In upstream regulators, upregulation of RICTOR, CPT1B, LARP1, and sirolimus suggested the alteration of mTOR/Akt and metabolism; the downregulation of LH, CREB1, MLXIPL, NGF, and TEAD1 suggested the disruption of hormone homeostasis; and downregulation of BCR, TCR complex, CD40, MYC, and MYCN suggested immune dysregulation and cellular hemostasis. A graphic summary connected the increased inflammatory response, growth factors, and decreased mTORC2 metabolism and reduced neuroprotective molecules such as CPE [24]. RNA‐seq analyses of PTZ‐induced epilepsy in rats via GO and KEGG enrichment revealed several hub genes and signaling pathways, including antiapoptosis as a mechanism of the anticonvulsant agent Q808 [33]. Both KEGG and IPA could provide potentially important molecular events. Interestingly, in our follow‐up studies in normal mice, IPA analysis of DEGs from HFD and Diazepam treatment generated opposite pathway changes compared with PTZ‐induced seizures [21], fortifying the beneficial effects of HFD.

Consistent to graphic network summary (Figure 4), PTZ‐induced kindling mice showed increased expression of proinflammatory markers [25, 26], BDNF/TrkB pathway [26, 34], GABA transporter and ion channel [26, 27], and apoptosis‐related genes [28]. As anticipated, diazepam and HFD recipes attenuated the expression of inflammatory marker *TNFα* and *NF-κB,* PTZ‐upregulated *TrkB* and *Bdnf*, and GABA transporter *Slc6a1 (GAT-1)* and ion channel *Trpm2*. The apoptosis‐related genes *parp1* and *Bnip3* increased by PTZ were also attenuated, consistent with the anticonvulsive effects.

Increased expression of “Immediate early genes”, such as *Fos, FosB* [16, 35], *Ptg2, Egr1* [36, 37], *Gadd45g, Btg, Arc,* and *Nr4a1* [38, 39], is frequently observed in the PTZ kindling model, and was evident in the present study in RNA‐Seq and qRT‐PCR analysis. The expression of these genes were significantly decreased after diazepam and original HFD, and attenuated or tended to decrease after reduced and nonfermented recipes, but were less effective after the half dose of reduced HFD.

Microbiota‐gut‐brain axis plays an important role in the pathogenesis of epilepsy [29]. In the PTZ kindling model, diazepam exerted antiepileptic effects via the modulation of gut microbiota composition and *α*‐diversity [18], the carbonic anhydrase inhibitor topiramate, together with *Lactobacillus johnsonii*, reduced the susceptibility to PTZ‐induced seizures [40], and the antiepilepsy effects of diosgenin in PTZ kindling mice are mediated through gut microbiota [19], whereas deletion of gut microbiota by a cocktail of antibiotics abolished the anticonvulsant effects of basil oil, with increased F/bacteroidetes ratio [17]. In the present study, the treatments slightly affected gut microbiota composition at the phylum and family levels, and the PTZ‐increased disruptions in four‐selected ASVs were ameliorated by diazepam and HFD to various extents. The modulation of *Lachnospiraceae* by HFD was also implicated in HFD against cerebral ischemic stroke [2], and PTZ‐increased ASV230 was also decreased by HFD in PTZ kindling mice and in normal mice [21]. Overall, these data imply the modulation of gut microbiota could be one of the mechanisms of the protection of HFD.

Nonfermented recipe was also effective in producing anticonvulsive effects in PTZ kindling mice, but also produced some anxiolytic effects in normal mice, although it was not as good as the original and reduced recipes, suggesting “fermentation” is an important process for HFD preparation. Chemical composition analysis was performed on nonfermented HFD and “Yaomu” [8, 9], and 51 differential ingredients between fermented and nonfermented HFD were identified [8]. It should also be noted that “*Yaomu*” fermentation not only affects liver gene expression [7], but also could enhance the beneficial effects of HFD in the treatment of ischemic stroke [8], consistent with the better antiepileptic effects observed in the present study.

It should also be mentioned that original and reduced HFD are safe in acute and chronic toxicity studies [1] and in pharmacological studies [2, 7, 8], either via garage or through drug‐containing feed [3, 10]. It should also be noted that other herbs or ingredients in the formulae could protect cinnabar toxicity as exemplified by *Angong Niuhuang* Wan [41, 42], *Zhusha Ansheng* Wan [43], and many others. Indeed, in the Pharmacopeia of China, many formulas contain a high amount of cinnabar, such as *Shayao* (11%), *Xiao′er Jindan Pian* (12%), *Xiao′er Jingfeng San* (12%), *Jufang Zhibao San* (12%), *Niuhuang Qiangjin San* (17%), *ZSASW* (24%), *Hongling San* (24%), and *Biwen San* (44%) as listed in the Pharmacopeia of China [5]. Nonetheless, caution should be taken to avoid the long‐term use of these cinnabar‐ and realgar‐containing traditional medicines, and the benefit and risk should be balanced.

There are several limitations in the study: first, only male mice were used; female animals will be considered in future studies; second, the specific bioactive molecules of HFD responsible for the antiepileptic effects require further investigation.

## 5. Conclusion

The present study demonstrated HFD recipes were effective against PTZ‐induced seizures in mice, probably through the suppression of PTZ‐induced brain gene expression and modulation of PTZ‐induced gut microbiota dysbiosis, providing pharmacological basis for its clinical use for epilepsy.

**Nomenclature**
PTZ: pentylenetetrazol
HFD: Hua‐Feng‐Dan
RNA‐Seq: RNA sequencing
PCA: principal component analysis
DEG: differentially expressed gene
IPA: Ingenuity Pathway Analysis

## Author Contributions

Jin Li and Jie Liu conceived and designed the study. Wen‐Ke Li carried out the main experiments and prepared the manuscript draft. Jin Li, Wen‐Ke Li, Bo Liu, and Shang‐Fu Xu participated in data curation, investigation, validation, and visualization. Shang‐Fu Xu, Jin Li, and Jing‐Shan Shi provided resources, fund acquisition, and administration. All authors reviewed and edited the manuscript.

## Funding

This study was supported by the National Natural Science Foundation of China (Grant No. 82360859, 82460879, U1812403, 82560832).

## Disclosure

All authors approved the final version of the manuscript.

## Ethics Statement

The experimental protocol followed the Chinese Animal Use and Welfare Guidelines and was approved by the Institutional Animal Care and Use Committee of Zunyi Medical University (ZMU21‐2403‐054).

## Conflicts of Interest

The authors declare no conflicts of interest.

## Supporting Information

Additional supporting information can be found online in the Supporting Information section.

## Supporting information


**Supporting Information 1** Table S1: Primer sequences for qPCR.

**Supporting Information 2** Table S2: 2D‐cluster of DEGs.

## Data Availability

The RNA‐seq and 16S rRNA‐seq data generated in the study are publicly available. This data can be found here: https://zenodo.org/records/14913055, Version 1.
